# Regulatory T cells in skin regeneration and wound healing

**DOI:** 10.1186/s40779-023-00484-6

**Published:** 2023-10-23

**Authors:** Samuel Knoedler, Leonard Knoedler, Martin Kauke-Navarro, Yuval Rinkevich, Gabriel Hundeshagen, Leila Harhaus, Ulrich Kneser, Bohdan Pomahac, Dennis P. Orgill, Adriana C. Panayi

**Affiliations:** 1 Division of Plastic Surgery, Department of Surgery, Brigham and Women’s Hospital, Harvard Medical School, Boston, MA 02115 USA; 2 Division of Plastic Surgery, Department of Surgery, Yale New Haven Hospital, Yale School of Medicine, New Haven, CT 06510 USA; 3 Institute of Regenerative Biology and Medicine, Helmholtz Zentrum München, Munich, 85764 Germany; 4 Department of Hand, Plastic and Reconstructive Surgery, Microsurgery, Burn Trauma Center, BG Trauma Center Ludwigshafen, University of Heidelberg, Ludwigshafen, 67071 Germany

**Keywords:** Regulatory T cells (Tregs), Wound healing, Wound repair, Skin injury, Skin regeneration

## Abstract

As the body’s integumentary system, the skin is vulnerable to injuries. The subsequent wound healing processes aim to restore dermal and epidermal integrity and functionality. To this end, multiple tissue-resident cells and recruited immune cells cooperate to efficiently repair the injured tissue. Such temporally- and spatially-coordinated interplay necessitates tight regulation to prevent collateral damage such as overshooting immune responses and excessive inflammation. In this context, regulatory T cells (Tregs) hold a key role in balancing immune homeostasis and mediating cutaneous wound healing. A comprehensive understanding of Tregs’ multifaceted field of activity may help decipher wound pathologies and, ultimately, establish new treatment modalities. Herein, we review the role of Tregs in orchestrating the regeneration of skin adnexa and catalyzing healthy wound repair. Further, we discuss how Tregs operate during fibrosis, keloidosis, and scarring.

## Background

Skin is the largest and most versatile organ in the human body. As the integumentary system of the body, it forms a barrier between the host and the external environment that enables defense against foreign pathogens. This function of the cutaneous system is mediated by a myriad of highly specialized immune cells. In interaction with other non-immune cells, such immune cell subsets ensure the functionality and integrity of the skin layers [1].

Given its large surface area, the skin is susceptible to injury. Loss of tissue is then repaired in a complex process that involves tissue-resident immune, stromal, and epithelial cells, as well as infiltrating immune cells [2]. Skin wound healing is a finely balanced biological process that can be subdivided into four stages (hemostasis, inflammation, proliferation, and remodeling) and involves a number of stage-specific molecular pathways [3]. The repair process can be interrupted at any stage of wound healing, which may lead to wound infection, wound dehiscence, non-healing and chronic wounds, and excessive scarring [4–6]. The maintenance of tissue homeostasis and proper wound healing is, therefore, a critical process that can be disturbed by a plethora of external and internal factors. Specifically, the timely orchestration of pro- and anti-inflammatory responses, based on the activation and inhibition of various cell types, such as tissue-resident cells and different types of immune cells, can significantly impact wound repair [7]. Regulatory T cells (Tregs) play a pivotal role in this context. Tregs are commonly defined as CD4^+^CD25^+^Foxp3^+^CD127^−^ T cells and their main function is to maintain self-tolerance of the immune system, with T cells dampening, suppressing, or stimulating the activity of other immune cells and resident fibroblasts [8–11]. Depending on their site of action, Tregs mediate tissue-specific functions, aiming to uphold or restore immune balance [12, 13]. To this end and within their broad functionality and operationality, Tregs are able to adopt unique features of skin cells and contribute to the skin’s role as an immune barrier.

When the barrier function of the skin is breached, inflammatory wound healing is initiated, whereby the immune system takes over a major role in orchestrating the subsequent phases of skin repair [14]. Cutaneous wound healing is a temporally- and spatially-synchronized cascade of cellular and immune responses dedicated to re-establishing skin integrity and function [3]. As such, this wound healing process calls for fine-tuned regulation and control. There is a mounting body of evidence that Tregs may act as powerbrokers in this context, maintaining skin immune homeostasis and mediating cutaneous wound healing [15–17].

While researchers agree on the abundance of Tregs in distinct dermal layers, the exact involvement of Tregs in cutaneous wound healing remains to be elucidated [18]. Therefore, in this review, we shed light on the enigma of Tregs and their specialized role in skin regeneration and wound healing.

## Anatomy of human skin

**Fig. 1:**
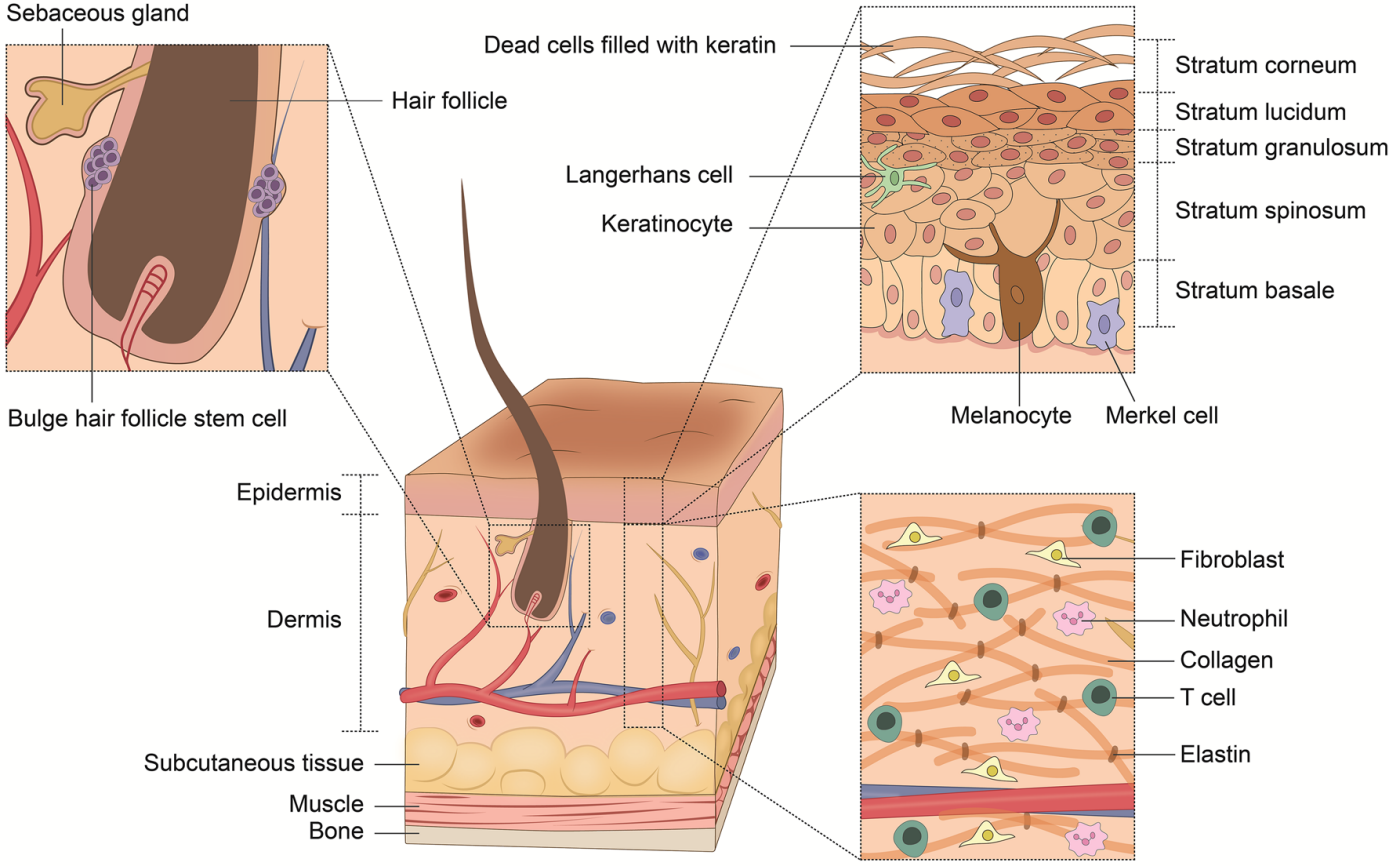
Cross-section of the human skin. The human skin is composed of the epidermis, dermis and subcutaneous tissue. The epidermis is a multilayered keratinizing squamous epithelium with keratinocytes representing the most common cell type in this skin layer. The stratum basale and stratum spinosum also harbor Merkel cells, melanocytes, T cells, and Langerhans cells. These four cell populations account for about 10% of epidermal cells. The dermis is home to immune cells, such as granulocytes and lymphocytes, and connective tissue-forming cells (fibroblasts). While collagen fibers and elastic fibers form the cellular fundament, blood and lymph vessels are also interwoven throughout this layer. As a form of elongated invagination, the hair follicle reaches out into the dermis. Sebaceous glands are located in close proximity to the hair follicle and its stem cells

The mammalian skin consists of three main layers: the epidermis, the dermis, and the subcutaneous tissue, including the fascia (Fig. 1). These epidermal and connective tissue layers protect against mechanical and chemical harm and repel pathogenic invaders [19]. If the physical barrier is penetrated, skin-resident immune and stromal cells cross-communicate and initiate wound healing immediately after the injury [3].

The main cellular components of the epidermis are keratinocytes, which produce keratins (intra-cellular fibrillar proteins). In interaction with the extracellular matrix, these proteins ensure the necessary resistance of the epidermis to tensile forces [20]. Characteristically, keratinocytes account for approximately 90% of the epidermal cell population and act as a building material for skin adnexa, such as hair follicles (HF) [21, 22]. As part of wound healing during the re-epithelialization phase, epidermal basal layer cells proliferate to give rise to neo-keratinocytes [23]. The remaining epidermal cell subsets comprise antigen-presenting dendritic Langerhans cells, dendritic epidermal T cells, melanocytes as pigment-producing cells, and Merkel cells for pressure detection and tactile sensation [24].

The dermis hosts sweat glands, sebaceous glands, and HF, with the latter following a cycle of growth, shedding, and regeneration phases, primarily mediated by stem cells located in bulges around the hair follicle. Together with the subcutaneous tissue, the dermis also harbors blood vessels, nerve endings, and lymphatic vessels [25, 26]. Tregs are attracted by hair follicle-secreted chemokines and, thus, localize in the dermis and epidermis to this perifollicular area – neighboring with further immune cells, such as effector T cells, macrophages, and antigen-presenting cells [27]. As a collagen-rich, connective tissue skin layer, the dermis is also home to an abundance of stromal cells. Dermal and subcutaneous fibroblasts produce elastin, collagen, and other components of the extracellular matrix, with the hydrologic capacity of hyaluronic acid contributing to tissue hydration, essential for maintaining cutaneous integrity during healing and repair processes [28–30]. Hence, the dermis and subcutaneous connective tissues, such as the fascia, are of fundamental importance for the maintenance and restoration of skin integrity, both in a steady state and during wound healing. Invaginated into the dermis, the subcutaneous tissue, also called the subcutis, represents the lowermost layer of the human integumentary system. The subcutis consists of loose connective tissue, referred to as superficial fascia, and the hypodermis layer rich in adipocytes [31, 32]. Generally, the subcutaneous tissue is highly vascularized and includes a network of neurons and lymphatics, forming migrant ducts for immune cells [33]. Indeed, the subcutis acts as the deepest layer of the skin’s immune system and buffers superficial inflammatory processes [34]. To this end, subcutaneous adipose and fascia tissue harbor macrophages with an M2 phenotype and CD4^+^ T cells with T helper cell type 2 (Th2) polarizations, indicating primarily anti-inflammatory and regulatory functions [35]. Accordingly, macrophages of the dermal white adipose tissue have been shown to facilitate skin repair [36, 37]. Recent studies also reported the presence of distinct Tregs subsets in subcutaneous tissue, the role of which may involve the promotion of thermogenesis and adipocyte beiging [38, 39]. In sum, the subcutaneous tissue not only serves as a shifting layer and heat/energy storage but also fulfills immunological protective functions and regulates tissue repair [40].

## Cutaneous wound healing

**Fig. 2:**
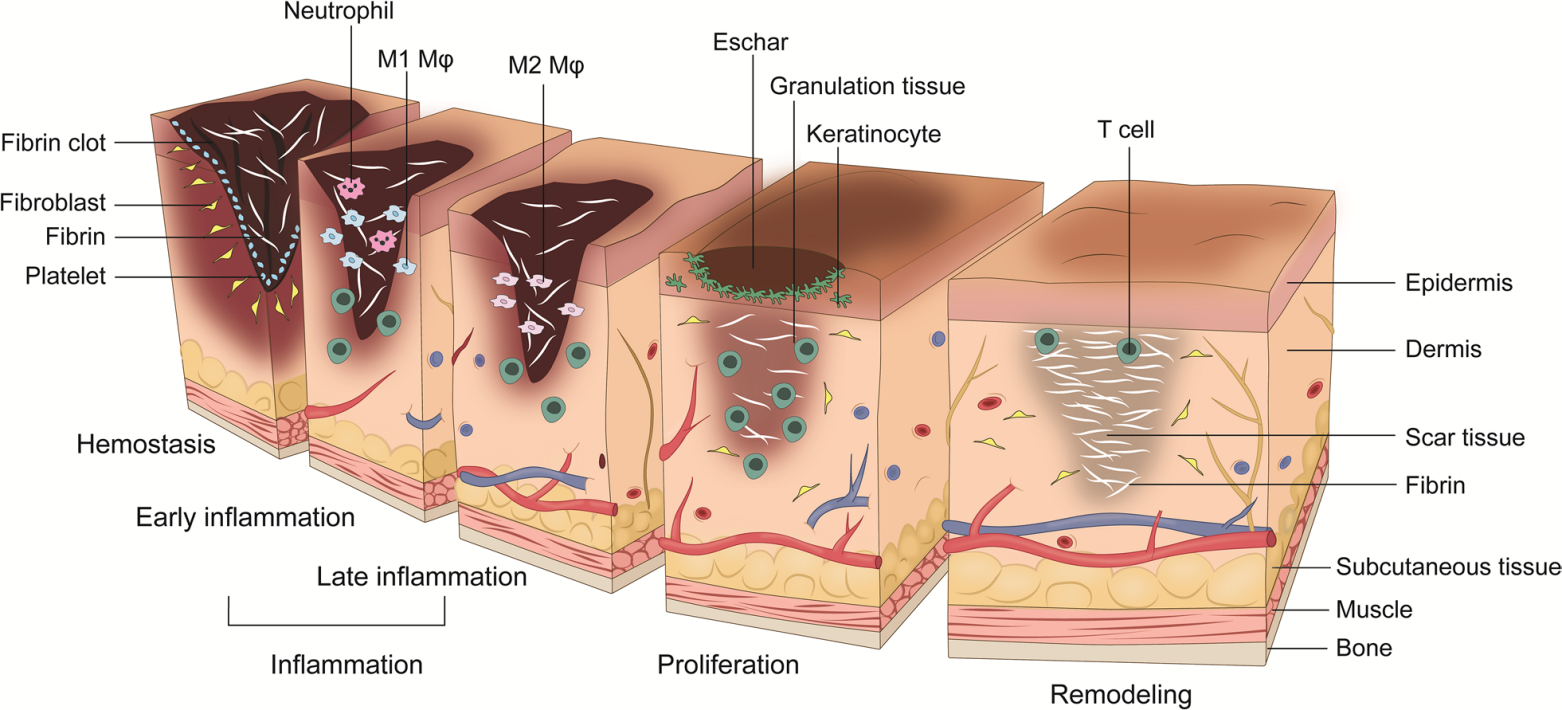
Wound healing stages. Wound healing is a biological process consisting of four overlapping phases (hemostasis, inflammation, proliferation and remodeling). Immediately after the injury, platelets migrate to the wound site. The release of chemical signaling reagents triggers the activation and polymerization of fibrin leading to the adhesion of platelets. Thus, fibrin (fibrin clot) and coagulated blood accumulate in the wound site. During the inflammation phase, monocytes migrate into the wound tissue, differentiate into tissue macrophages, and subsequently phagocytose the blood coagulum. Growth factors are released into the wound site, stimulating cells to proliferate. As a result, by secreting collagen and fibronectin, fibroblasts form a provisional extracellular matrix. Simultaneously, the squamous epithelium of the skin is renewed via the ingrowth of new epithelial cells from the wound edges (epithelialization). Finally, the final scar tissue is formed, which lacks hair follicles as well as sebaceous and sweat glands. Mφ macrophage

Skin wound healing is a highly complex process subdivided into four sequential but overlapping stages (Fig. 2).

### Initial hemostasis

Reactive vasoconstriction of injured blood vessels and activation of the coagulation cascade lead to hemostasis as the organism’s first response to skin injury [2]. Platelets bind to the subendothelial collagen and fibronectin via the glycoprotein Ib (GPIb) and GPIc/IIa receptor with the involvement of the von Willebrand factor [41]. Subsequently, in the course of this platelet activation, more surface receptors, such as GPIIb/IIIa, are expressed, and degranulation of α- and β-granules, including vascular endothelial growth factor (VEGF), platelet-derived growth factor (PDGF), and transforming growth factor-beta (TGF-β), occurs [3, 42]. Of note, in the existing scientific literature, TGF-β is defined as both a multifunctional growth factor and a pleiotropic cytokine [43, 44]. The wound gap is then closed provisionally by aggregation of platelets with fibrinogen as a binding agent [2]. Downstream, platelets, erythrocytes, and leukocytes entangle in the resulting network of fibrin, nectin, and thrombospondin. This coagulum, known as red thrombus, is a scaffold that serves as a guide for the migration of epidermal and endothelial cells, fibroblasts, granulocytes, and monocytes [2, 45].

### Inflammation

In addition, growth factors released from the blood clot, such as TGF-β and PDGF, as well as cytokines, such as tumor necrosis factor-alpha (TNF-α), interleukin-1 (IL-1), and IL-8, mediate the chemotaxis of additional immune cells [2, 3, 46]. More specifically, neutrophilic granulocytes, which prevent and clear wound infections by phagocytosis of bacteria and removal of devitalized tissue, and monocytes follow chemotactic stimuli [3]. Early inflammation is, therefore, characterized by diapedesis of these leukocytes, with a peak 24–48 h after skin injury [2]. Monocytes will become activated (tissue) macrophages and phagocytose tissue debris while secreting cytokines and chemokines that trigger the influx and activity of other immune cells, primarily T helper cells and natural killer cells [3] Beyond this role as wound cleansers and mediators of extracellular matrix remodeling, macrophages also regulate the proliferation of fibroblasts and endothelial cells and, therefore, play an integral role in the formation of granulation tissue [3]. In this setting, they secrete cytokines with angiogenic and profibrotic effects, including TGF-α, TGF-β, TNF-α, PDGF, VEGF, IL-1, IL-6, insulin-like growth factor 1 (IGF-1), basic fibroblast growth factor (bFGF/FGF2) [2, 3]. Macrophages in the wound area reach their maximum concentration after 72–96 h, orchestrating the late inflammation and transition to the proliferation phase [47, 48] in dual functioning, they regulate a sensitive balance between tissue formation and degradation. It is noteworthy that the site of macrophage action is mainly focused on the upper dermis, whereas T cells tend to reside in the deep dermis of the wound edge [49]. This relatively low concentration of T cells in the first day after wounding increases markedly from day 7 and peaks on day 14, with the highest detectable levels in the phases of late inflammation and early/mid proliferation [49]. T cells may be particularly involved in the mechanisms of healthy tissue repair and dermal scar formation. As early as in this inflammatory phase, the process of wound contraction begins. The granulation tissue is traversed by a meshwork of α-smooth muscle actin (α-SMA)-expressing myofibroblasts [50]. Through cell-cell bonds between these myofibroblasts mediated by cadherins and connexin gap junction, intertwining of the (myo)fibroblasts with collagen fibers, and alignment of the collagen fibers, the wound edges are gradually contracted [51, 52].

### Proliferation and tissue formation

Proliferation is characterized by the induction of angiogenesis and simultaneous sprouting of fibroblasts along the fibrin network into the tissue defect (induced by IL-1, PDGF, and TGF-β), forming collagen, and releasing hyaluronic acid and chondroitin sulfate as matrix components of the newly formed cell- and vessel-rich granulation tissue [2, 46]. Endothelial cells are stimulated by bFGF and VEGF to produce and establish novel vascular structures [53]. Generally, angiogenesis is driven by growth factors (especially VEGF) secreted by macrophages, endothelial cells, and blood cells and stimulated by the local wound environment, i.e., tissue acidosis and hypoxic conditions [2]. Keratinocytes are triggered to migrate and expand both by local growth factors, e.g., TGF-α, TGF-β, and keratinocyte growth factor (KGF/FGF7), and mechanical factors (“free edge effect”), ultimately achieving re-epithelialization of the defect [3, 54]. For this purpose, with the involvement of contractile actin-myosin filaments, keratinocytes proliferate, migrate to the wound matrix, and gradually rebuild a continuous epithelial layer [55]. As soon as epithelial fronts converge, the mechanism of contact inhibition leads to cessation. Interestingly, no T cells are detectable in this newly re-formed epidermis [49]. At this stage, lymphocytes are predominantly localized in the papillary dermis in the vicinity of the hair follicle bulges.

Scar tissue formation and skin contraction are further achieved by the active transport of extracellular matrix from the fascia, deep beneath the wound edges [56, 57]. This transport of extracellular cell matrix (ECM) is mediated by swarm-like collective migration of a fibrogenic cell lineage termed as EPFs (for engrailed 1-lineage positive fibroblasts) [58]. Fascia transport by fascial fibroblasts is a key step in wound closure and scar formation. Genetic depletion of fascial fibroblasts or placement of a film barrier beneath the skin halt fascia transport into wounds and results in chronic open wounds that fail to heal [51, 56]. In general, any deviation from the finely calibrated healing cascade may lead to chronic non-healing wounds. Characterized by impaired and/or delayed wound repair, such wounds do not properly progress through the healthy stages of wound healing. The chronicity of wounds may be driven by a wide array of etiologies, including vascular disorders, diabetes mellitus, and immune system dysregulation (e.g., due to T cell imbalance) [6, 14, 59–61].

### Remodeling

The final repair phase is hallmarked by remodeling, during which immature type III collagen is replaced by more resistant type I collagen [62]. This collagen shift is mediated by matrix metalloproteinases and their counterparts, namely tissue inhibitors of metalloproteinases, in a finely balanced process wherein macrophages exert a crucial control and regulatory role [2, 3, 62]. At the end of the remodeling processes and maturation of the collagenous fibers, a cell- and vessel-depleted scar without any skin adnexa is left [63]. Macrophages, fibroblasts, and endothelial cells previously present in the wound environment perish by apoptosis and the capillary growth ceases, with a standstill and reduction of cell- and blood vessel-rarefication [64]. Finally, α-SMA expressing myofibroblasts – driven by TGF-β – lash the wound edges tightly together, and the wound surface shrinks, leaving a pale light-colored scar in which the collagen layers are no longer complexly intertwined but parallelly arranged [64].

The organism’s immune response to skin injuries, ranging from superficial abrasions to deeply penetrating wounds, and the incipient process of wound repair necessitate regulation. In this context, Tregs act as mediators between immune tolerance and excessive inflammatory reaction. Recently, novel tissue reparative functions of these cells have been uncovered, rendering Tregs to (hidden) background managers of skin regeneration and wound healing. Therefore, this review aims to summarize and portray the role of Tregs in healthy and injured skin.

## Definition of Tregs

**Fig. 3:**
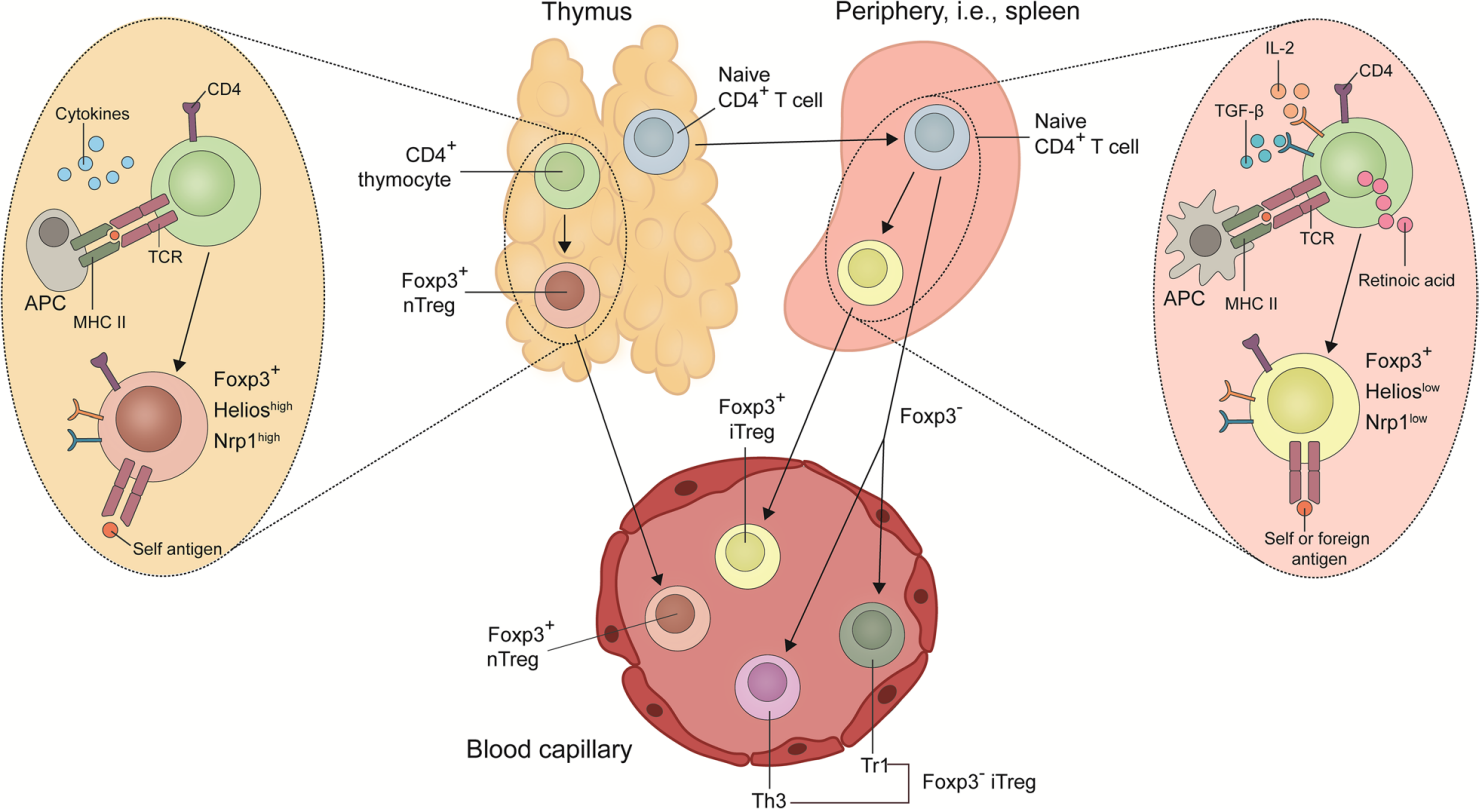
Nature of regulatory T cells (Tregs). Representing a specialized subset of T lymphocytes, Tregs commonly exhibit a distinctive genetic phenotype with upregulation of genes encoding signature proteins, such as FOXP3. Essentially, Tregs originate from two sources: (1) the majority of Tregs arise during thymic T cell maturation (nTregs). They constitute a steady population carrying CD4 and expressing T cell receptors (TCRs) that enable them to recognize self-antigens. (2) Tregs can also be generated in the periphery from conventional CD4^+^ T cells upon antigenic exposure and stimulated by high levels of TGF-β, retinoic acid, and IL-10. Such iTregs are equipped with TCRs that can also detect foreign antigens. Different Treg subsets including nTregs, iTregs and Foxp3^−^ Tregs circulate in the bloodstream. APC antigen presenting cell, CD4 cluster of differentiation 4, Foxp3 forkhead box protein P3, IL-2 interleukin-2, iTregs induced Tregs, MHC II major histocompatibility complex II, nTregs natural Tregs, TGF-β tumor growth factor-beta

Tregs represent a specialized subset of T lymphocytes, accounting for approximately 5–10% of all CD4^+^ T cells in the human body [65]. Tregs exhibit a characteristic genetic phenotype with upregulation of genes coding for various proteins, such as cytotoxic T lymphocyte protein 4 (CTLA-4), IL-2 receptor subunit-α (CD25), and most notably forkhead box protein P3 (Foxp3) [66]. Currently, CD4^+^Foxp3^+^CD25^+^CD127^−^ T cells are thought to be the most prevalent Treg phenotype [9, 10, 67]. Depending on their origin in the thymus or periphery, two types of CD4^+^ Tregs are differentiated [67, 68]. It is worth mentioning that the T cell receptor (TCR)-repertoire differs between these two Treg populations (Fig. 3) [69].

### Thymus-derived Tregs (t-Tregs)

The t-Tregs develop during thymic T cell maturation upon high avidity recognition of self-antigens. They carry CD4 as well as CD25 and express the L-selectin receptor CD26L and CTLA-4 [70, 71]. Representing a stable population with an abundance of TCRs that detect self-antigens, this Treg population is able to inhibit autoreactive T cells, and, thus, suppress autoimmune reactions [72]. To this end, t-Tregs secrete a wide array of immunosuppressive molecules, including IL-4, IL-10, interferon gamma (IFN-γ), and TGF-β [73–76]. In vitro studies indicated that CD4^+^CD25^+^ Tregs are anergic themselves and can only be activated to proliferate in combination with IL-2 and TCR stimulation [77, 78].

### Peripherally-derived Tregs (p-Tregs)

The p-Tregs are generated in the periphery from conventional CD4^+^ T cells in response to antigen exposure and stimulated by high levels of TGF-β, retinoic acid, IL-10, and TNF-α [79–81]. In contrast to t-Tregs, p-Tregs also express TCRs that recognize foreign antigens [82]. By targeting such foreign antigens, one of the main tasks of p-Tregs is to block inflammatory responses against commensal microorganisms [83–86]. They exert this suppressive function by secreting a specific cytokine pattern, especially IL-10, IL-5, IFN-γ, and TGF-β [75, 87, 88].

Of note, Tregs that are CD8^+^ have also been identified. This CD8^+^ Tregs population exhibits limited cytotoxicity and represents mostly memory cells inhibiting tumor-associated T cell activation of antigen-presenting cells via secretion of IL-10. They can be generated in vitro by stimulation with CD40-activated plasmacytoid dendritic cells and share phenotypic features with p-Tregs [89]. To this date, the available body of evidence suggests that Tregs isolated from the peripheral blood for therapeutic purposes are most likely a combination of the two subtypes p-Tregs and t-Tregs [90].

In humans, mutations in the gene encoding Foxp3 (master regulator of Tregs) result in a rapidly fatal autoimmune disease known as immune dysregulation, polyendocrinopathy, enteropathy X-linked (IPEX) syndrome. Likewise, scurfy mice, which are deficient in functional Tregs due to a frameshift mutation in the *Foxp3* gene, suffer from an analogous disorder with overwhelming systemic autoimmunity [91]. In both humans and mice, such immune dysregulation is reflected in cutaneous symptoms [91, 92]. Accordingly, qualitative and quantitative disorders of Tregs have been implicated in the pathogenesis of skin diseases. The spectrum of dermatological pathologies associated with Tregs ranges from alopecia areata (AA), vitiligo, and psoriasis over atopic dermatitis, pemphigus, and pemphigoid diseases to cutaneous lupus erythematosus and scleroderma [12, 93]. The fact that dysregulation of Tregs is associated with numerous diseases of cutaneous autoimmunity and chronic skin inflammation underscores the indispensable role of Tregs in maintaining and restoring the immune balance in the skin. In general, Tregs suppress the activation of the immune system and thereby regulate the immune self-tolerance [70]. Specifically in the skin, they not only prevent disease development but also orchestrate the regeneration of skin adnexa and catalyze wound healing.

## Tregs in healthy skin

As the barrier between the inner organism and outside influences, the skin must be capable of mounting effective and strong immune responses. Wounds are natural entry points for invading pathogens, making the skin all the more important as a competent immune organ. However, such ability of targeted antigen defence inevitably entails the risk of aberrant and dysregulated immune reactions and, thus, calls for sophisticated regulation and control mechanisms. The exceptionally high baseline population of Tregs in the skin, which physiologically accounts for 20–80% of CD4^+^ T cells in this tissue, is, therefore, not surprising [12].

### Migration and skin-homing of Tregs

Scharschmidt et al. [16] have demonstrated that, in murine skin, Tregs accumulate primarily during a defined one-week window of neonatal development. From postnatal day 6–13, a wave of TCR-αβ expressing T cells (of which 80% were highly activated Tregs) migrated into the skin. Inhibition of overall lymphocyte population migration during this period resulted in a pooling of Tregs in the thymus, suggesting a thymic origin of these later skin-resident Tregs. Interestingly, the researchers found this Treg accumulation to be skin-specific and not caused by a systemic or global increase in thymic efflux. In fact, no comparable Treg enrichment was observed in either intestinal lamina propria or skin-draining lymph nodes. In addition, Scharschmidt et al. [94] noted a critical role of hair follicle morphogenesis and commensal microbiota during this flushing process of Tregs into the skin, with both leading to increased expression of the CC-chemokine ligand 20 (CCL20) [ligand of the CC-chemokine receptor 6 (CCR6)] from hair follicle epithelial cells. Accordingly, CCR6-expressing Tregs migrated preferentially from the neonatal thymus into the skin during early life phases. Thus, the interaction of the receptor CCR6 expressed by skin Tregs and its ligand CCL20 mediated the Treg recruitment in the skin in vitro and in vivo. Germ-free neonatal mice harbored reduced levels of skin Tregs, indicating the relevance of commensal microbes in establishing and augmenting Treg build-up in the skin. However, the extent to which these findings from the animal model can be transferred to the human organism and whether an abrupt wave with seeding of Tregs into skin layers also occurs in human neonatal life have yet to be determined. While the exact role of the CCL20-CCR6 pathway in the skin-homing of human Tregs remains to be elucidated, Cook et al. [95] documented the mediating function of this axis in the migration of Tregs into *Helicobacter pylori*-infected human gastric mucosa.

Investigating the developmental trajectories of IL-33 receptor (ST2)^+^ skin Tregs in a murine model, Delacher et al. [96] found two precursor stages, which also resided in lymphoid tissues prior to skin seeding. Specifically, the researchers identified Klrg1^−^Nfil3(GFP)^+^ and Klrg1^+^Nfil3(GFP)^+^ as lymphoid precursor stages for ST2^+^ skin Tregs. Single-cell RNA-sequencing data, RNA-velocity analyses, transfer experiments, and chromatin profiling revealing a stepwise increase in chromatin accessibility suggested a sequential differentiation of Klrg1^−^Nfil3(GFP)^+^ via Klrg1^+^Nfil3(GFP)^+^ cells into tissue-resident Klrg1^+^Nfil3(GFP)^+^ST2^+^ skin Tregs. Interestingly, similar to the observations of Scharschmidt et al. [94], Delacher et al. [96] documented a peak in precursor output on postnatal day 10, echoing in a more than 22-fold increase in Klrg1^+^Nfil3(GFP)^+^ ST2^+^ Tregs per gram skin within 5 day (between neonatal day 10 and 15). Further analyses revealed the transcription factor basic leucine zipper transcription factor, ATF-like (BATF) to drive the cell differentiation from the precursor stage into the skin Tregs’ phenotype by orchestrating a molecular tissue-specific precursor program. The absence of this key transcription factor BATF resulted in a deficiency of progenitor cell differentiation and, subsequently, mature ST2^+^ Tregs in the skin [96]. More recently, the Delacher group [97] validated the significance of BATF in human Tregs, with BATF postulated to induce both tissue differentiation and repair programs. In this study, they used single-cell chromatin accessibility in combination with gene expression profiling and TCR fate mapping to characterize a population of tissue-like Tregs in human peripheral blood expressing BATF, CCR8, and human leukocyte antigen (HLA) DR isotype. Such BATF^+^CCR8^+^ Tregs from healthy skin shared common properties with nonlymphoid T follicular helper-like (Tfh-like) cells, with the induction of a Tfh-like differentiation program in naïve human Tregs promoting tissue Treg regenerative capacity (including wound healing potential).

Hirahara et al. [98] provided evidence that a vast majority of CD4^+^CD25^high^Foxp3^+^ Tregs in the peripheral circulation express the skin-homing receptors CCR4 (nearly 100%), cutaneous lymphocyte-associated antigen (CLA, a carbohydrate modification of P-selectin glycoprotein ligand-1; 80%), and CCR6 (73%). The researchers demonstrated the functional competence of these molecules by CD62E ligand activity of CLA^+^ Tregs and verifiable chemotactic responses to the CCR4 ligands CCL22 and CCL17, as well as to the CCR6 ligand CCL20. In this context, CCR4 holds a special role: animal studies have revealed that, in the absence of CCR4 expression, the accumulation of Tregs was significantly impaired, with such CCR4-deficient mice even showing lymphocytic infiltration and severe inflammatory diseases in the skin [99, 100]. Further, CCR5 and CCR8 with their ligands CCL4/CCL5 and CCL1, respectively, are discussed as skin-homing receptors [101]. In fact, Delacher et al. [97] documented that blood memory CCR8^+^ Tregs may represent putative skin Treg precursors (i.e., a developmental path from blood naïve via blood memory to dedicated CCR8^+^ tissue Tregs) and/or recirculating skin Tregs, thereby serving as a reserve pool of tissue-engaged Tregs with specific gene expression, TCRs, and wound repair potential. The expression of the enzyme α-1,3-fucosyltransferase VII (FuT7), which generates the determinants of E-selectin and P-selectin binding, is also relevant for Tregs trafficking to healthy, non-inflamed skin [102]. According to Dudda et al. [103], the absence of FuT7 resulted in significantly reduced accumulation of Tregs in murine skin, eventually causing severe cutaneous inflammation. Such skin inflammatory reactions (spontaneously or reactive) could also be observed in mice with RAR-related orphan receptor alpha (RORα) or GATA binding protein 3 (GATA3)-deficient Tregs, respectively [104, 105]. While the impact of GATA3 on skin-homing receptor expression remains unclear (impaired Treg stability due to a reduction in Foxp3 expression in the absence of GATA3 is hypothesized to be the culprit of poor inflammatory control), RORα deficiency altered the expression of genes involved in Treg skin migration, such as CCR6. In any case, GATA3 is essential for proper functioning of Tregs. GATA3^+^ Tregs have been found to express the IL-33 receptor ST2 [106, 107]. This tissue-derived nuclear cytokine is typically secreted by cells under stress and in agony [108]. Activated via the IL-33-ST2 pathway, GATA3^+^ Tregs can then initiate mending responses.

### Localization and maintenance of Tregs

In general, it is important to distinguish between two subtypes of Tregs: first, skin-resident Tregs, which are already present in the tissue and respond rapidly to injury, and second, Tregs that migrate from the bloodstream to the injury site and require prolonged activation and polarization time [27].

In healthy humans, skin-resident Tregs preferentially localize to the epidermis and dermis around HF, a well-known reservoir of commensal microbiota and stem cells [109]. Accordingly, Sanchez Rodriguez et al. [110] showed that human Tregs could be detected primarily in regions with high HF density, i.e., face and scalp. This affinity of Tregs to HF could also be established in uninflamed murine skin: utilizing multiphoton microscopy of healthy abdominal skin, clustering of Tregs around HF was observed [111]. In accordance with these findings, Tregs are generally scarce in the region of interfollicular dermis and epidermis.

Using an ovalbumin-expressing mouse model, Rosenblum et al. [112] first identified a Treg population that persisted in the skin long-time after antigen clearance and was able to mitigate skin inflammation upon repeated antigen exposure. Such memory Tregs have also been detected in human skin, with comparatively low proliferative index and non-migratory, anergic properties under steady-state conditions [110]. In detailed phenotypic analyses, over 95% of Tregs in the skin of healthy adults expressed protein tyrosine phosphatase receptor type C (PTPRC or CD45RO), suggesting previous or current antigen exposure. In addition, high levels of the memory markers CD27 and B cell lymphoma-2 (BCL-2) were found in these Tregs. Taken together, these data indicate an activated effector memory phenotype of almost all human skin Tregs exposed to skin-related antigens and gradually accumulating over time. Therefore, Ali et al. [18] speculated that Tregs in non-inflamed, healthy skin predominantly represent a “slow cycling tissue‐resident population”. Interestingly, human fetal skin harbored fewer Tregs (defined as CD45^+^CD3^+^CD4^+^Foxp3^+^) that mainly lacked CD45RO expression, suggesting that the majority of these cells are exposed to antigens after or during birth [110].

Of note, memory Tregs in human skin expressed low levels of CD127 (IL-7Rα), whereas the comparable murine Treg subset (memory Tregs) showed increased CD127 expression and required IL-7 for maintenance in case of antigen absence [110, 113]. As a rationale for this dependence on IL-7, the research team led by Gratz et al. [113] suspected a dynamic adaptive response by memory Tregs to ensure survival in tissues with relatively low local IL-2 concentrations, while IL-2 plays a minor role in the maintenance of memory Tregs in the skin, IL-2 is a key factor in generating memory Tregs from naïve CD4^+^ T cell precursors in vivo.

Besides the role of specific cytokines, the interaction with other cell types is also relevant for the maintenance of skin-resident Tregs: dermal fibroblasts emerged as necessary for the survival of Tregs in the skin [78]. Further, in the presence of IL-15, contact with such fibroblasts triggered Tregs, isolated from normal human skin, to proliferate. It is worth noting that IL-2 addition, although not required, slightly increased Treg proliferation. Interestingly, this Treg expansion was antigen- and costimulation-independent [78]. Langerhans cells are inactive dendritic cells in the stratum spinosum of the epidermis, which normally serve for antigen presentation [114]. Such epidermal Langerhans cells are able to induce activation and proliferation of human skin Tregs in vitro and in vivo [115]. Strikingly, since this Langerhans cell-mediated proliferation could be completely inhibited by antibodies against major histocompatibility complex (MHC) class II or CD80-86, it likely proceeds in an antigen-dependent manner. Antibodies against the cytokines IL-2 and Il-15 also blocked such expansion. Another subset of dendritic cells in the dermis of human skin, namely vitamin D_3_-inducible, IL-10-producing, CD141^+^ dendritic cells, were found to mediate Tregs suppressing cutaneous inflammation in vivo [116]. The association between dendritic cells and Tregs was investigated in a murine skin model, wherein short-wave ultraviolet B (UVB) irradiation led to a significant proliferation of Tregs lasting for 14 d [117]. In the dermis of UVB-exposed mice, Tregs formed clusters with dendritic cells. Notably, the expanded Tregs in UVB-treated mice expressed cutaneous homing receptors, such as CCR4 and CD103, and were, thus, able to migrate to other non-UVB-irradiated skin areas.

Briefly, multiple signaling routes and attractants pave the way for the entrance and residence of Tregs. As a result, Tregs are uniquely poised to uphold the immune homeostasis in the skin and intervene rapidly in the event of immune interference, inflammation, and injury. The skin-specific armamentarium of Tregs primarily includes the abilities to regenerate HF as well as to mediate wound repair and healing (Table 1) [15, 17, 109, 118–130].

**Table 1:** Overview of relevant scientific findings in the field of regulatory T cells in hair regeneration and cutaneous wound healing

Field	References	Main findings
Tregs in hair regeneration	Ali et al. [109]	Tregs mediate HF cycling by enhancing HFSC differentiation and proliferation. Expression of the Notch ligand Jagged-1 on Tregs was identified as a major mechanism underlying Tregs’ promotion of the HFSC function and HF regeneration. Immunosuppressive properties of Tregs (i.e., suppression of the INF-γ signaling pathway) did not restore HFSC activation. A three-fold higher concentration of Tregs was detectable in telogen skin relative to the anagen state. Treg-depleted mice showed less than 20% of hair regrowth compared to the control group two weeks after depilation
	Petukhova et al. [118]	In patients with AA, single nucleotide polymorphisms in genes controlling the nature of Tregs were found. These genes were *IL-2*/*IL-21*, *IL-2RA* (IL-2 receptor A; CD25), *Eos* (also known as Ikaros family zinc finger 4; IKZF4), and *CTLA-4* as well as* NOTCH-4*
	Zöller et al. [119], Hamed et al. [120], Mukhatayev et al. [121]	In both human studies and murine models of AA, both quality and quantity of Tregs were found to be reduced
	Castela et al. [122]	Recruitment of Tregs in lesional AA skin promoted via low-dose subcutaneous IL-2 administration has been shown to be effective in AA treatment, with successful hair regeneration being observed in 80% of cases. Results were maintained at 6 months without serious complications
	Lee et al. [123]	Expanding Tregs in murine skin alone did not trigger hair growth and failed to suppress autoreactive CD8^+^ T cells to reverse established AA. Upon intradermal injection of IL-2/anti-IL-2-antibody-complex, selective proliferation of Jagged-1^+^ Tregs was noted but immune privilege around hair follicles was not restored. Tregs were unable to promote hair growth by inducing telogen-to-anagen transitions
Tregs in healthy wound healing	Mathur et al. [124]	In a murine wound model, after depletion of Tregs, IL-17-producing CD4^+^ T cells proliferated, leading to an increased expression of the neutrophil chemoattractant CXCL5. The hyperinflammatory CXCL5-IL-17 response blocked HFSC differentiation and impaired their migration into the interfollicular epidermis, ultimately hindering skin repair. Tregs were found to control the local inflammatory environment by suppressing the CXCL5-IL-17 axis, thereby promoting HFSC differentiation during the reestablishment of the skin barrier
	Truong et al. [125]	HFSCs may orchestrate the de novo generation of extrathymic Tregs during wound healing. HFSCs expressing CD80 deliver a stimulatory signal to effector CD4^+^ T cells infiltrating into the wound, stimulating them to differentiate into Tregs. These Tregs can protect HFSC from collateral damage induced by inflammatory wound neutrophils
	Nosbaum et al. [15]	Tregs facilitate skin wound healing and mitigate wound-associated inflammation. Seven day after full-thickness wounding, the density of Tregs in the wounded skin peaked, reaching levels that were 20-fold higher than at baseline. Specifically, activated Tregs expressing high levels of CD25, CTLA-4, ICOS, and epidermal growth factor receptor (EGFR) (in contrast to Tregs of skin-draining lymph nodes) were observed among the accumulated Tregs. When these Tregs were ablated, there was a significant reduction in re-epithelialization and wound closure, along with an increase in IFN-γ-producing T cells and a buildup of proinflammatory macrophages. Specific deletion of EGFR led to delayed wound closure and significant cellular shift early during the inflammatory phase of wound healing with a decrease of Tregs and an increase in proinflammatory macrophages. Treg-depleting after the inflammatory phase did not influence wound healing kinetics markedly
	Haertel et al. [126]	Treg-depleted mice showed impaired wound healing (i.e., decreased wound contraction, delayed re-epithelization, and compromised vessel maturation). Mechanistically, Treg depletion resulted in a significant increase in IL-4 levels combined with an overexpression of T-box transcription factor 21 (TBX21 or T-bet)^+^ and GATA-3^+^ αβ T cells. In addition, an expansion of IL-17A- and IFN-γ-producing CD4^+^ and CD4^−^ αβ T cells was noted. In short, depletion of Tregs was associated with an accumulation of specific αβ T cell population, with alternations in the cytokine (micro)milieu interfering with the healthy wound healing program
Influence of UVB on healing and repair functionality of Tregs	Shime et al. [127]	After UVB irradiation, Tregs expanded in the skin, featuring a unique T cell receptor repertoire and expressing genes associated with wound healing, including those encoding proenkephalin (PENK, an opioid precursor) and amphiregulin (AREG, an EGFR ligand). In a skin explant assay, Treg-derived PENK and AREG promoted the outgrowth of keratinocytes. In addition, UVB-expanded skin Tregs promoted wound healing in vivo. Conversely, the depletion of Tregs exacerbated inflammation in the UVB-exposed skin of mice and impaired wound healing. More precisely, the absence of Tregs was reflected in an increased expression of IL-1β, IFN-γ, TNF-α, and accumulation of Ly6C^high^ proinflammatory macrophages, and clinically by an aggravation of ear swelling induced by UVB irradiation
Tregs in scarring and fibrosis	Kalekar et al. [17]	Acute and chronic depletion of Tregs in mice led to upregulated profibrotic gene expression, uncontrolled skin fibroblast activation, and dermal fibrosis. Upon Treg-specific deletion of GATA-3, the quantity of skin-infiltrating proinflammatory macrophages and neutrophils did not change markedly, whereas the levels of IL-13 and IL-4-producing Th2 cells along with dermal fibroblast activation increased significantly, ultimately leading to skin fibrosis characterized by dense collagen, increased skin thickness, and reduced dermal adipose tissue. Taken together, Tregs were found to serve as key regulators of skin fibroblast activation and, hence, suppression of profibrotic skin immune responses, expressing a dedicated transcriptional program mediated (in part) by GATA-3
	Murao et al. [128]	Co-culturing keloid fibroblasts with Treg-enriched CD4^+^ T cells elevated profibrotic IL-6 but decreased mRNA expression of type I collagen and TGF-β – findings indicative of an anti-fibrotic and protective function of Tregs in the pathogenesis of keloids. The ratio of Tregs/CD4^+^ T cells in keloid tissue was reduced in comparison to other inflammatory skin disorders and rather similar to healthy skin. In sum, a local imbalance of Tregs may underlie keloidosis and could provide a therapeutic avenue for keloid management in the future
	Chen et al. [129]	Compared to healthy skin, Treg-associated gene expression, including IL-10 and TGF-β, was significantly upregulated in human keloid tissue. In addition, a correlation between the frequency of Tregs (i.e., the expression of Foxp3) and collagen expression was noted. These findings suggest that Treg dysregulation is limited to the keloid lesion sites. In fibrocytes incubated with activated Tregs (i.e., those pre-stimulated via anti-CD3/CD28 antibodies), the collagen expression was increased. This observation was more pronounced in keloid patients than in non-keloid controls and was postulated to require the secretion of TGF-β. In conclusion, the researchers deduced a link between collagen overexpression/imbalance and Treg dysregulation in keloids
Tregs in chronic and diabetic wound healing	Barros et al. [130]	In a full-thickness murine diabetic wound model, CCR4 and Tregs were found to negatively affect wound healing. Reduced Treg migration into wounded skin was seen in CCR4 knockout diabetic mice. When neutralizing antibodies against the CCR4 ligands, CCL17 and CCL22, were applied, reduced Treg levels and improved wound healing were noted in diabetic wildtype mice (despite worsened diabetes status) as compared to untreated control mice. Neutralization of CCL17/22 also resulted in a decrease in Foxp3 transcripts in the wound bed. Treg depletion via anti-CD25 administration led to improved wound healing kinetics in diabetic mice, indicating a negative effect of Tregs during wound repair

*HF* hair follicle, *HFSC* hair follicle stem cell, *IFN-γ* interferon gamma, *AA* alopecia areata, *IL* interleukin, *CTLA-4* cytotoxic T lymphocyte protein 4, *CXCL5* CXC motif chemokine 5, *ICOS* inducible co-stimulator, *GATA-3* GATA binding protein 3, *Ly6C* lymphocyte antigen 6 complex, *TNF* tumor necrosis factor, *UVB* ultraviolet B, *TGF* transforming growth factor, *Foxp3* forkhead box protein P3, *CCR* CC-chemokine receptor, *CCL* CC-chemokine ligand

## Tregs in hair regeneration

As mentioned above, the preferential location of skin Tregs is around HFs. HF act as anchors for hair and undergo cyclic activity. The hair cycle ranges from hair growth in the anagen phase through its shedding in the catagen phase to the HF regeneration in the telogen phase (resting phase) [131]. The bulge of HF harbors a major subset of stem cells (HFSC) with indispensable roles, including the orchestration of a smooth transition between phases of the HF cycle [109]. This cell population can be identified by the expression of their molecular markers, such as B lymphocyte-induced maturation protein-1 (Blimp1; sebaceous gland stem cells), GLI family zinc finger-1 (Gli1), and leucine-rich repeat-containing G-protein coupled receptor 6 (Lgr6; lower isthmus stem cells), as well as Lgr5 and CD34 [132]. Notably, a subpopulation of Tregs in telogen skin tissue was localized in close vicinity to the HFSC niche [109]. This local association was mirrored at the functional level: Ali et al. [109] found Tregs to promote HFSC differentiation and proliferation, ultimately mediating HF cycling. One of the underlying mechanisms by which Tregs enhance HFSC function (in the absence of skin injury) was identified in a Treg expression of the Notch ligand Jagged-1 (JAG-1). The authors also noted that the immunosuppressive properties of Tregs are not the driving factors for HFSC activation. Suppression of the INF-γ signaling pathway, which is considered a hallmark function of Tregs, did not restore HFSC activation [15, 133]. This was reproducible for genetic deletion, as well as antibody neutralization of Tregs together with CD4^+^ T cells, CD8^+^ T cells, protein gamma response 1 (Gr-1)^+^ neutrophils, and CD11c^+^ myeloid cells [109].

Using immunophenotypic profiling, Ali et al. [109] pinpointed the specific HF cycle phase during which the quantity and activation of Tregs increase abruptly in murine skin: a three-fold higher concentration of Tregs was detectable in telogen skin (compared to anagen status). Further analyses of the proliferation markers Kiel-67 (ki-67), CD25, inducible co-stimulator (ICOS), glucocorticoid-induced TNFR-related protein (GITR), and CTLA-4 revealed a significantly higher activity level of Tregs in the telogen phase. Consistent with these findings revealing the importance of Tregs for HF regeneration, it was found that, in Treg-depleted mice, two weeks after depilation, less than 20% of hair regrowth compared to the control group was seen, with a substantially reduced anagen induction [109].

AA is an autoimmune disease and the most common cause of inflammation-related hair loss [134]. The autoimmunity in AA is site-specific and mainly mediated by natural killer group 2D (NKG2D)-expressing autoreactive CD8^+^ T cells targeting HF [135]. A genome-wide association study in AA identified single nucleotide polymorphisms in genes controlling the nature of Tregs [118]. Among the genes with significant association to AA, *IL-2*/*IL-21*, IL-2 receptor A (*IL-2RA*; CD25), *Eos* (also known as Ikaros family zinc finger 4; IKZF4), and *CTLA-4* were found. Interestingly, *NOTCH-4* also appeared as a potential candidate gene. These findings indicate an association between Treg dysfunction and AA pathogenesis. Indeed, both human studies and murine C3H/HeJ models of AA provided evidence for reduced quantity and quality of Tregs in patients suffering from AA [119–121]. Accordingly, Tregs’ inherent immunosuppressive function of pathogenic T cells has been shown to be impaired [136].

Nevertheless, there are controversies regarding the therapeutic value of Tregs in AA. In a prospective pilot study on 5 patients conducted by Castela et al. [122], recruitment of Tregs promoted by low-dose subcutaneous IL-2 administration was found to be effective in the treatment of severe AA. In 80% of cases, successful hair regeneration was observed, which the investigators attributed to an accumulation of Tregs within the diseased scalp skin. Notably, the effects were maintained at 6 months, with an even increased hair (re)growth. Contrary to these promising findings, in a C3H/HeJ mouse model of AA, the expansion of Tregs failed to reverse established AA [123]. Although intradermal injection of IL-2/anti-IL-2 antibody complex (IL-2c) yielded selective proliferation of Jagged-1^+^ Tregs, the immune privilege around HF could not be restored. Likewise, the expanded skin Tregs were unable to promote hair growth by inducing telogen-to-anagen transitions. Therefore, future studies are needed to thoroughly investigate the therapeutic potential of Tregs in AA and, ultimately, leverage Tregs’ impact on hair regeneration.

## Tregs in wound repair and healing

### Tregs in healthy cutaneous wound healing

While typically involved in hair regeneration, HFSC may also differentiate into epithelial cells, promoting skin repair [137]. By facilitating the restoration of the cutaneous barrier, HFSC then contributes to the prevention of wound infection and water loss. As mentioned above, Tregs are able to mediate the customization of HFSC – even into such reparative epithelial cells – and thereby promote epidermal regeneration after injury. Using a tape-stripping model of epidermal abrasion, Mathur et al. [124] demonstrated that in the absence of Tregs, IL-17-producing CD4^+^ T cells (i.e., Th17 cells) proliferate, resulting in increased expression of the neutrophil chemoattractant CXC motif chemokine 5 (CXCL5). Such hyperinflammatory CXCL5-IL-17 response blocks HFSC from differentiating and impairs their migration (for subsequent barrier repair) into the interfollicular epidermis. During all four stages of wound repair, Tregs control the local inflammatory environment by suppressing the CXCL5-IL-17 axis. In short, Tregs facilitate HFSC plasticity, which is essential for the prompt re-establishment of the skin barrier following trauma. Vice versa, preliminary data reported by Truong et al. [125] points toward skin stem cells orchestrating de novo generation of extrathymic Tregs to establish a temporary protective niche during wound healing. The authors used a model of cutaneous wound healing in which HFSCs repaired the wound site. They suggested that HFSCs expressing CD80 deliver a stimulatory signal to effector CD4^+^ T cells which infiltrate into the wound. This signaling triggered effector CD4^+^ T cells to differentiate into Tregs. These Tregs, in turn, can protect HFSC from collateral damage induced by inflammatory wound neutrophils.

In the event of injury, Tregs proliferate, exploiting further levers to promote and facilitate skin healing. Upon full-thickness wounding, a subpopulation of highly activated Tregs has been shown to accumulate in murine skin [15]. Specifically, 7 d after injury (i.e., after the resolution of the inflammatory phase and onset of the proliferative stage), the density of Tregs peaked, with a 20-fold higher level than at baseline. Tregs from secondary lymphoid organs predominantly migrated into the injured skin. Interestingly, among the accumulated Tregs, high expression levels of CD25, CTLA-4, and ICOS were noted. Specific ablation of these Tregs resulted in significantly reduced re-epithelialization and attenuated wound closure, with increased formation of granulation tissue and eschar in the wound bed. Such deletion early after injury during the inflammatory stage led to an increase of IFN-γ-producing T cells and an IFN-γ-driven accumulation of proinflammatory macrophages. In the persistence of these lymphocyte antigen 6 complex (Ly6C)^high^ macrophages throughout the entire course of wound closure, Nosbaum et al. [15] suspect a major culprit for delayed wound healing. Of note, Treg-depleting after the inflammatory phase, i.e., between day 5 to 12, did not markedly interfere with wound healing kinetics.

**Fig. 4:**
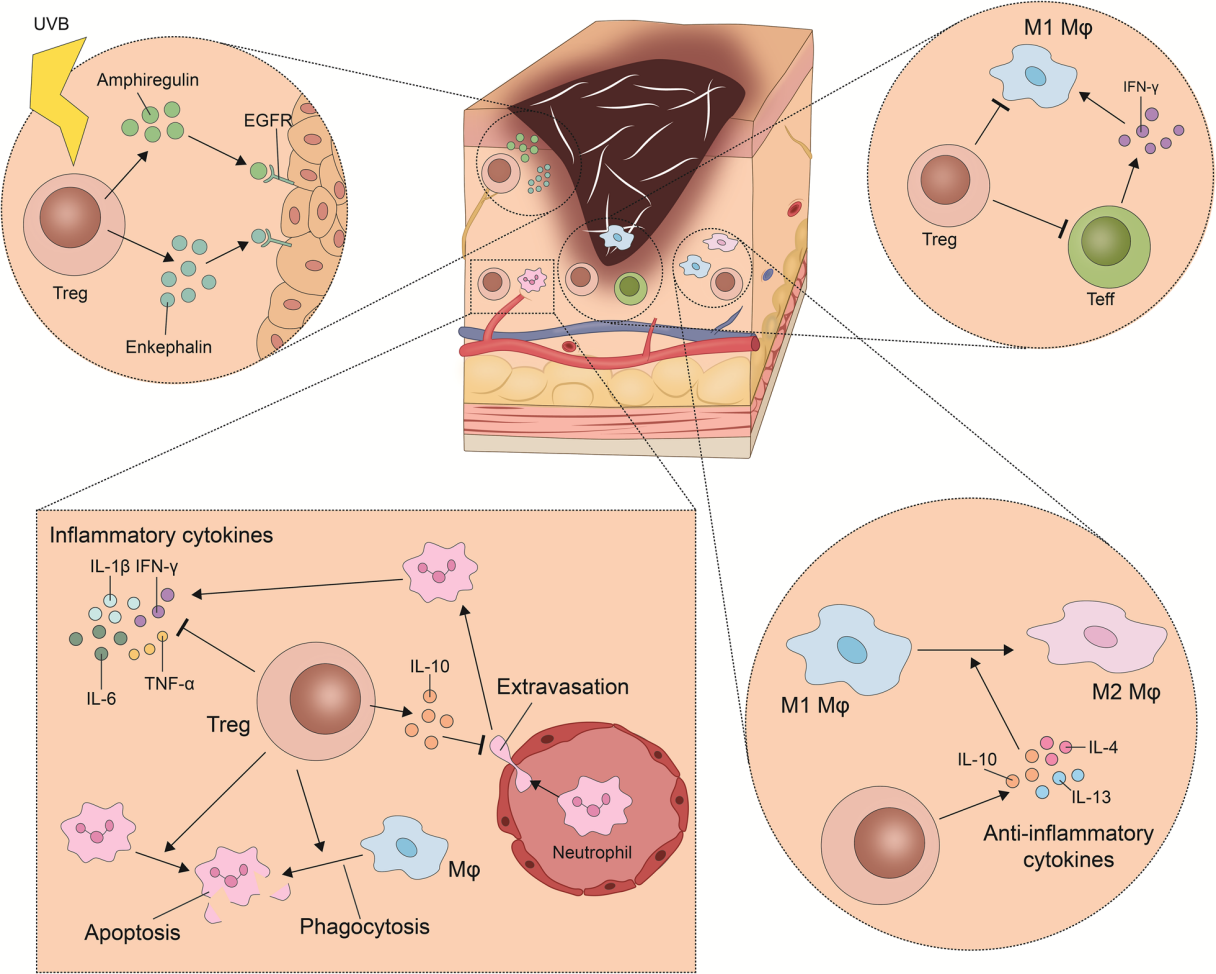
Cellular cross-talks of regulatory T cells (Tregs) in the skin. In response to UVB radiation, Tregs secrete amphiregulin and enkephalin which bind to EGFR receptors and promote keratinocyte outgrowth. Tregs can suppress T effector cells (Teff) and M1 macrophages, as well as hinder the differentiation of M1 macrophages into M2 macrophages. Tregs can also secrete IL-10 hindering the extravasation of neutrophils and the production of proinflammatory cytokines such as TNF-α and IL-6. EGFR epidermal growth factor receptor, IFN interferon, IL-1β interleukin-1β, TNF tumor necrosis factor, UVB ultraviolet B, Mφ macrophage

Furthermore, Nosbaum et al. [15] reported a considerable expression of epidermal growth factor receptor (EGFR) on Tregs in inflamed skin. In contrast, Tregs of skin-draining lymph nodes did not express EGFR at any time point post-wounding. Remarkably, lineage-specific deletion of EGFR led to delayed wound closure and significant cellular shift early during the inflammatory phase of wound healing: while the accumulation of Tregs in wounded skin decreased, an increase in proinflammatory macrophages was seen in Foxp3^cre^EGFR^fl/fl^ mice. In summary, these findings substantiate the concept that Tregs facilitate cutaneous wound healing and mitigate wound-associated inflammation, with the EGFR pathway playing a central role (Fig. 4). In the field of wound healing, EGFR is known to orchestrate a plethora of biological responses [138–140]. Its pleiotropic functions include catalyzing re-epithelialization, driving keratinocyte proliferation, and stimulating angiogenesis. Via downstream signaling pathways, such as phosphoinositide-3-kinases (PI3K)/Akt kinase (Akt)/mechanistic target of rapamycin (mTOR) route, EGFR may operate as an accelerator of wound healing and conveyor of dermal maturation [141]. Epidermal growth factor (EGF), transforming growth factor-alpha (TGF-α), heparin-binding EGF-like growth factor (BG-EGF), epigen, and amphiregulin (AREG) are listed among the ligands with binding ability to EGFR [142]. Stimulated by IL-18 and IL-33, Tregs in murine skin can generate AREG [143]. This protein enhances the proliferation of keratinocytes and, by triggering TGF-β activation on mesenchymal stromal cells (pericytes), AREG helps to restore the vascular integrity in injured tissues [144]. Hence, these findings indicate that reparative measures in interstitial connective tissue may also be facilitated by AREG-EGFR signaling [145].

Wounding stimulates Tregs to express a wide array of further growth factors. In zebrafish, Tregs drive retinal repair by secreting IGF-1 [146]. Similarly, after lung injury, Tregs express KGF to facilitate alveolar regeneration by type 2 alveolar epithelial cells [147]. Of note, dendritic epidermal T cells release both IGF-1 and KGF to promote epidermal regeneration after skin injury. Therefore, one may hypothesize that Tregs also reap the benefits of these peptides during cutaneous wound healing. However, leveraging the positive pro-healing effects of these growth factors has not yet been verified for skin Tregs.

The ability of Tregs to significantly regulate the expression of other molecules upon wounding was demonstrated in another murine wound experiment [126]. Treg-depletion led to a sharp increase in IL-4 levels in conjunction with an increased proportion of T-box transcription factor 21 (TBX21 or T-bet; inducer of *IFN-γ* gene transcription)^+^, RORγt (regulator of IL-17A expression)^+^, and GATA-3^+^ αβ T cells. In line with these findings, an accumulation of IL-17A- and IFN-γ-producing CD4^+^ and CD4^−^ αβ T cells was also detectable. Haertel et al. [126] attributed the elevated IL-4 levels mainly to the abundance of GATA-3^+^ cells. In essence, this recent study reiterated the necessity of functional Tregs for healthy wound healing: Treg depletion led to a delay in re-epithelialization, a reduction in wound contraction, and impaired vascular maturation. It is, therefore, not surprising that, at day 7 after wounding (a time point by which normally the remodeling phase is initiated), Treg-depleted mice still showed increased keratinocyte proliferation rates – a cellular correlate reflecting the need for continued re-epithelialization processes. However, in contrast to the findings by Nosbaum et al. [15], no increase in CD11b^high^Ly6C^high^ proinflammatory macrophages was seen [126]. Interestingly, in this study, the researchers also investigated the involvement of Tregs in accelerated wound repair of mice overexpressing the proteohormone activin. In fact, in the absence of Tregs, this accelerated wound healing of activin-transgenic mice was nullified. Taken together, these results are a testament to the far-reaching and essential regulatory influence of Tregs during the endogenous wound healing program, as their absence is echoed in cell population shifts and alterations in the cytokine (micro)milieu which compromised healthy wound repair.

### The influence of UVB on healing and repair functionality of Tregs

As mentioned above, UVB exposure may induce the expansion of Tregs in the skin, suggesting a potential therapeutic benefit of UVB in cutaneous disorders [117]. In a recent study, Shime et al. [127] investigated the functional characteristics of such UVB-expanded Tregs in the skin. After UVB irradiation, Tregs were shown to expand markedly, featuring a unique TCR repertoire and expressing a gene set associated with wound healing. Using RNA-sequencing, among the UVB-expanded Tregs in murine skin, the increases in gene expression of encoding proenkephalin (PENK, an opioid precursor) and AREG were found to be most prominent. Subsequent analyses revealed the neuropeptide signaling route (with PENK being the main core-enriched gene) to be the most-enriched pathway in UVB skin Tregs. A PENK-derived neuropeptide, methionine enkephalin (Met-ENK), is known to induce opioid δ-receptors on keratinocytes, accelerating and promoting wound repair [148]. When added to skin explants from naïve mice, both Met-ENK and AREG significantly increased keratinocyte outgrowth [127]. Likewise, the administration of UVB-expanded skin Tregs into such skin explants of naïve mice also resulted in the promotion of keratinocyte growth. Strikingly, when applying naltrindole, a specific δ-opioid receptor antagonist, or anti-AREG neutralizing antibody in the ex vivo skin explant assay, no keratinocyte proliferation was detectable. These results highlighted that skin Tregs generate PENK and AREG following UVB exposure to enhance keratinocyte outgrowth, thus implying a healing function of Tregs in UVB-irritated murine skin.

In addition, the research team revealed that Treg-depletion exacerbated inflammation in the UVB-exposed skin of mice [127]. At the molecular-cellular level, the absence of Tregs was reflected in an increased expression of IL-1β, IFN-γ, TNF-α, and accumulation of Ly6C^high^ proinflammatory macrophages, and clinically-macroscopically, an aggravation of ear swelling induced by UVB irradiation emerged. From this inflammatory response, once again, the far-reaching ramifications and regulatory capacity of skin Tregs – this time in the setting of UVB – can be deduced. More so, Tregs were also identified as the driving force of the wound healing effect following UVB irradiation in a murine skin wound model whereby full-thickness wounds were made on day 6 after UVB exposure, i.e., at a stage when Treg expansion had already occurred [127]. UVB irradiation in Treg-sufficient mice promoted wound healing not only compared with Treg-depleted mice but also with control naïve mice, yielding relative elongation of epidermal tongues at day 5 after wounding. In addition, the keratinocyte proliferation (a hallmark of the proliferation and tissue formation phase) was found to be significantly increased in UVB-irradiated mice as measured in skin explants harvested 6 d after UVB exposure. In contrast, in Treg-depleted mice, UVB irradiation failed to stimulate wound healing and a delay in wound healing kinetics was noted compared to both control naïve mice and UVB-irradiated Treg-sufficient mice.

### The role of Tregs in scarring and fibrosis

Fibrosis is the umbrella term for two processes in the field of wound healing: on the one hand, “fibrosis” can be used to refer to the physiological deposition of connective tissue leading to scar formation. On the other hand, fibrosis is also a synonym for the pathological excess deposition of fibrous tissue [149]. Molecularly, the pathophysiology of fibrosis is similar to the physiological process of scarring [150]: upon tissue damage, macrophages are activated, releasing multiple cytokines and growth factors such as PDGF and TGF-β. These agents, in turn, initiate a variety of cellular signaling pathways (including Akt/mTOR), thus inducing the proliferation of fibroblasts and their siblings myofibroblasts [151]. Through the subsequent production of glycosaminoglycans and collagen, the extracellular matrix densifies [152]. However, these normally physiologic cascades can escalate, culminating in irreversible and exuberant fibrosis of pathologic severity. To prevent such excessive and overshooting accumulation of extracellular matrix components, the process of fibrosis requires finely balanced regulation. Tregs play a unique role in this regulation by bridging the thin line between physiological fibrosis (as part of healthy scarring) and excessive pathological fibrosis.

Prima vista paradoxically, Tregs have been shown to produce TGF-β, which may exert both anti-inflammatory and fibrogenic functions under certain conditions [153]. Yet, using RNA sequencing, Kalekar et al. [17] demonstrated that skin-resident Tregs generate comparatively less of this potentially profibrotic cytokine. In addition, the researchers hypothesized that Tregs in the skin may even act as “TGF-β sinks”, thereby thwarting the cytokine in its activation of fibroblasts [17]. Indeed, Tregs rather regulate fibrosis and fibroblast activation, as an in-depth investigation of Tregs’ role in the pathogenesis of fibrosis shed light on their anti-fibrotic orientation [17]. After depleting Tregs in mice, the α-SMA (a key marker of fibrotic responses)-expressing myofibroblasts increased fivefold within 5 d and profibrotic gene expression was markedly upregulated. Similarly, chronic reduction of Tregs also yielded a marked increment of such fibrogenic α-SMA-expressing dermal myofibroblasts, increased expression of profibrotic genes, and downregulation of antifibrotic genes. It is worth noting that, in this chronic setting, a significant decrease in subcutaneous dermal adipose tissue and an increase in dermal collagen density with markedly expanded dermal thickening were also found. These results imply a regulatory role of Tregs for skin fibrosis in steady-state and suggest that excessive scarring can be prevented through Tregs. Of note, the effects of acute and chronic Treg depletion were worsened in a bleomycin-induced murine model of skin sclerosis, underscoring the paramount importance of Tregs in pathological skin disorders.

Compared with other tissues, skin Tregs express relatively high levels of GATA-3, the main transcription factor for Th2 polarization [12, 154]. Therefore, it was theorized that GATA-3 may be responsible for mediating the aforementioned anti-fibrotic effects of Tregs in the skin. Subsequent Treg-specific deletion of GATA-3 revealed one reason for the abundant expression of this transcription factor in skin Tregs [17]: while the proportion of skin-infiltrating proinflammatory macrophages and neutrophils did not change appreciably in such treated mice, the percentages of IL-13 and IL-4-producing Th2 cells increased significantly and dermal fibroblast activation with clearly augmented α-SMA-expressing myofibroblasts skyrocketed. In parallel, collagen densified, cutaneous thickness rose, and dermal adipose tissue shrank. These distinct signs of skin fibrosis in mice deficient in GATA-3-expressing Tregs accentuate the significance of GATA-3 and highlight its key role in curbing fibroblast (over-)activation and dermal fibrosis. In sum, Kalekar et al. [17] provided evidence for the regulatory role of skin Tregs in the activation of fibroblasts and, thus, in the suppression of profibrotic skin immune responses, employing a specific transcriptional program mediated (partially) by GATA-3.

**Fig. 5:**
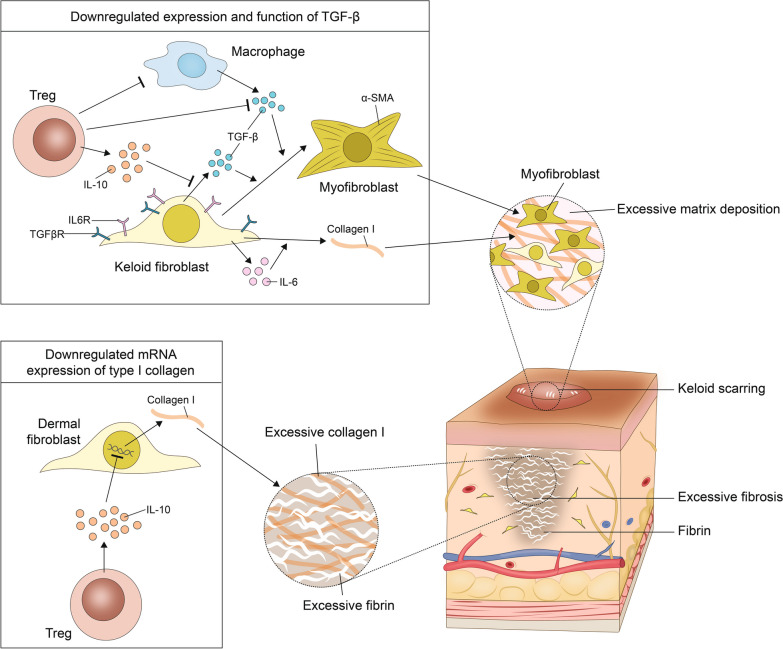
Regulatory T cells (Tregs) in keloid scarring. Tregs can downregulate the expression and cellular impact of TGF-β to target excessive matrix deposition as a hallmark of keloid scarring. Tregs can further reduce mRNA expression of type I collagen. Excessive collagen I and fibrin accumulations have been implicated with fibrosis as part of keloid formation. IL-10 interleukin-10, IL6R interleukin-6 receptor, TGFβR tumor growth factor beta receptor, α-SMA α-smooth muscle actin

Hyperproliferative fibroblasts and the excessive production/deposition of extracellular matrices may lead to uncontrolled growth of fibrotic tissue beyond the original wound lesion. The resulting keloid is a benign dermal tumor protruding above the skin level [155]. As a paradigm of excessive skin fibrosis, keloids are the result of dysregulated and dysfunctional wound healing [156]. A study from Japan aimed to decipher the role of Tregs in such keloids, with two main findings (Fig. 5) [128]: first, when coculturing keloid fibroblasts with Treg-enriched CD4^+^ T cells, the profibrotic and proinflammatory marker IL-6 was found to be significantly elevated. However, in this setting, the mRNA expression of type I collagen and TGF-β was significantly decreased, suggesting a protective and anti-fibrotic function of Tregs in the pathogenesis of keloids. Second, the ratio of Tregs/T cells in keloid tissue was not markedly increased and, hence, similar to the proportion in healthy skin. According to Murao et al. [128], this – compared to other inflammatory dermal disorders – relatively low Treg count may represent a potential avenue for the prevention and/or treatment of keloid fibrosis. Based on their findings, the researcher postulated that a local imbalance of Tregs may underlie the formation of keloids.

In another study investigating the involvement of Tregs in keloidosis, contradictory and startling results were reported, with (dysregulated) Tregs seemingly implicated in the pathogenic processes [129]. Specifically, both the abundance of Tregs and the transcript levels of the two Treg-associated cytokines (with pleiotropic functions) IL-10 and TGF-β1 were significantly elevated in keloid tissue compared with normal skin (and peripheral blood) [129, 157]. Moreover, in keloid tissue, a positive correlation was noted between the level of Foxp3 expression and the level of encoding type III collagen (type III collagen alpha 1, COL3A1) expression and the COL3A1/type I collagen alpha 1 (COL1A1) ratio. Whether dysregulated Tregs are the elicitors of collagen over-expression or whether collagen over-expression and the pro inflammatory environment seen with keloids along with an increase in macrophage presence triggers Tregs dysregulation remains to be elucidated. Regardless, when pre-stimulated with anti-CD3/CD28 antibodies, Tregs could increase in vitro collagen expression by fibrocytes, profibrotic cells of myeloid origin. Chen et al. [129] hypothesized that this increase in collagen expression is mediated by Treg production of TGF-β. Collectively, their data suggest that the dysregulation of Tregs is limited to the sites of keloid lesions and that there is an association between an imbalance of Tregs and collagen overexpression. Yet, this study left questions unanswered. Among other issues, immunohistochemical studies of cytokine expression are lacking, while possible confounding effects of the hair cycle precise localization of Tregs in keloid tissue remain unclear.

### Tregs in chronic and diabetic wound healing

Chronic non-healing wounds do not abide to the linear paradigm of the aforementioned healthy and sequential repair phases. Instead, such wounds are characterized by a delayed and/or dysregulated healing program that typically stagnates during the inflammatory or regenerative stages [158, 159]. Comorbidities such as vasculopathy and diabetes mellitus are believed to be the most common reasons triggering the pathological trajectory from timely and orderly repair to wound chronification [6, 59, 160–163]. Mechanistically, these causes typically overstimulate the inflammasome, thereby shifting the naturally sensitive balance between pro- and anti-inflammation. In this context of immune disbalance, Tregs may play an important regulatory role by curbing excess inflammation [164]. Yet, to date, Treg involvement in the pathogenesis and management of chronic and diabetic wounds remains to be fully elucidated, with preliminary evidence pointing toward a double-edged sword-like role of Tregs [27].

Holl et al. [165] and Rehak et al. [166] hypothesized that the systemic inflammation seen in patients with diabetes mellitus may impair the migration of Tregs while promoting the infiltration of inflammatory Th17 cells into the wound tissue. This deficit of inflammation-suppressing Tregs may contribute to an abnormally prolonged inflammatory response which results in non-healing wounds.

Leung et al. [167] found significantly reduced levels of Tregs in ischemic tissues from patients with diabetes mellitus compared to healthy normoglycemic individuals. In this study, the researchers also provided evidence that CD4^+^Foxp3^+^ Tregs can promote vascular regeneration and induce de novo sprouting angiogenesis after ischemic injury in diabetic mice. While the formation of new blood vessels from existing vasculature is considered a key step during the proliferative stage of healthy wound repair, it is also deemed a hallmark of chronic inflammation. Therefore, it is yet to be established to which extent Treg-mediated functional (re)vascularization (which has been validated in further studies) 1) translates to wound healing mechanisms in human skin and 2) triggers the chronification of wounds or promotes normal wound repair [168, 169].

A recently published study from Brazil takes a critical view of Tregs’ role in diabetic wound healing [130]. As outlined above, the chemokine receptor CCR4, with its ligands CCL17 and CCL22, has been implicated in the recruitment and activation of Tregs in the skin. The research team of Barros et al. [130] used a full-thickness murine diabetic wound model. Therein, CCR4 was found to impair wound healing, with relatively reduced levels of various cytokines (IL-1β, IL-6, IL-10, IL-12p70, and TNF-α) in diabetic CCR knockout (KO) mice (CCR4^−/−^), when compared to the CCR4^+/+^ diabetic control. Consistent with its role as a skin-homing receptor, CCR4-deficiency was associated with a reduction of Treg migration into wounded skin. Strikingly, when applying neutralizing antibodies against both CCR4 ligands, CCL17 and CCL22, diabetic wildtype mice showed faster wound healing than the non-treated counterpart. Yet, it is essential to emphasize that such anti-CCL17/22 injected mice suffered from hyperglycemia and showed a mortality rate of 50%. These findings suggest two intriguing conclusions: first, a protective role of Tregs in diabetogenesis, and second, despite worsened diabetes status, anti-CCL17/22 treated mice showed improved wound healing. Similar to CCR4 absence, neutralization of CCL17/22 also resulted in a decrease in Foxp3^+^ cells in the skin. Likewise, anti-CD25 administration (i.e., a common method of Treg-depletion) decreased Tregs’ count and led to improved wound healing kinetics in diabetic mice, underscoring a negative effect of Tregs during wound repair [170]. Collectively, these data point towards a detrimental role of Tregs in the wound healing of diabetic mice. In all three sub-conditions (CCR4 KO, anti-CCL17/22 treatment, and anti-CD25 treatment), Tregs frequency was reduced, and wound site healing was accelerated.

Overall, the paucity of evidence on Treg’s role in chronic and diabetic wounds calls for future studies. Understanding the involvement of Tregs in these wound pathologies is an integral step toward new therapeutic modalities in clinical management.

## Outlook

### Tregs in vascularized composite allotransplantation (VCA)

VCA, the transplantation of tissue blocks, such as the face from one donor to a genetically distinct recipient, has become the standard of care for selected patients with devastating facial defects [171]. For allograft survival, it is necessary that patients receive highly toxic but effective immunosuppressants, such as tacrolimus, mycophenolate mofetil (MMF), and steroids. Despite this, acute rejection of the skin portion of the allograft occurs in more than 85% of VCA patients within the first year and often multiple times thereafter [172, 173]. Given the non-life-saving nature of this procedure, it is particularly important to search for novel avenues that optimize the risk–benefit ratio of VCAs. In this context, the minimization of immunosuppressant-related side effects is of major interest, especially through the use of immunomodulatory cellular therapies [174]. Tregs are of unique value owing to their natural ability to suppress alloreactivity and maintain immune hemostasis, high efficacy, and potential to be engineered to target specific antigens [e.g., with chimeric antigen receptors (CAR)] [175, 176]. Approaches that improve recruitment and local potency, such as the design of microparticles that release T cell recruiting chemokines, systemic IL-2 administration, and Tregs engineered to carry IL-2 loaded nanoparticles have been investigated in preclinical models [177]. While the (preliminary) results of these studies appear promising, clinical translation is still pending.

In VCA, the transplanted units comprise a heterogenous set of tissues, including skin, mucosa, muscle, and vasculature. Therefore, this unique composition, with component-specific antigenicity, carries the risk of strong immune responses with potentially tissue-dependent mechanisms of rejection [178]. The design of CAR-Tregs may, therefore, represent an innovative strategy aimed to achieve tissue-specific immunoregulation. Similarly, topical administration of Tregs, amplification of donor hematopoietic engraftment for chimerism induction, and potentiation of skin-specific Treg function are also promising immunomodulatory strategies that warrant future studies investigating their applicability and utility in VCA [177].

### Genome editing in Tregs

Genome editing in Tregs is a broad field under intensive research. For the purpose of this review, we focused on clustered regularly interspaced short palindromic repeats (CRISPR)/CRISPR-associated protein 9 (Cas9), which represents a versatile tool for genome editing [179]. Specifically, CRISPR/Cas9 can be leveraged to knockout different target genes. Van Zeebroeck et al. [180] proposed the IL-6 receptor (IL6R; CD126) as a promising target because elevated IL-6 levels are found in different autoimmunopathies and have been implicated with impaired suppressive capacity in Tregs [180, 181]. The authors showed that IL-6-induced STAT3 signaling, which is associated with destabilization of Tregs’ function, was reduced in IL6R-KO Tregs. Yet, this promising target remains to be verified in vivo studies [182–184]. The purinergic receptor P2X7 (P2X7R) represents another gene target as it decreases the viability and suppressive function of murine Tregs [185]. Investigating experimental colitis in a murine model, Schenk et al. [186] found P2X7R-KO Tregs to express increased levels of the activation markers, TGF-β, and IL-10 [186, 187]. Further, the disruption of the programmed cell death protein 1 (PD-1) axis via genome editing has been linked to increased suppressive potency and longevity in murine Tregs [188]. CRISPR/Cas9 editing of the Fas cell surface death receptor (*FAS)* gene has been hypothesized to increase the viability of Tregs due to the pro-apoptotic signaling of *FAS* on Tregs’ cell fate [187]. Ring finger protein 20 (Rnf20) has also been recently discussed as a negative regulator of FOXP3 and seems to be another promising target for CRISPR/Cas9 genome editing [189]. While there is a wide panel of candidate target genes for CRISPR/Cas9 genome editing, future in vivo studies focusing on the cross-talk of Tregs and wound healing are needed.

### Future directions of Tregs research

Overall, the role of Tregs in wound healing is an emerging and rapidly evolving research field. Future efforts might harness the efficacy and versatility of CAR-equipped Tregs. The CAR construct is particularly intriguing as it may allow for more precise clinical use of Tregs by navigating the Tregs toward the site of their target antigen, which could alleviate systemic side effects [176]. In general, the potency of CAR-Tregs is well documented both in vitro and in vivo, with MacDonald et al. [190] providing evidence that CAR-Tregs are effective in controlling alloimmune-mediated rejection of human skin grafts. However, given the limited duration of experimental studies, the potentially detrimental long-term effects/sequelae of CAR-Tregs application, such as excessive Treg stimulation followed by Tregs exhaustion and cell death, remain to be investigated [27]. Beyond the potential implementation of CAR-Tregs in wound healing therapy, the application spectrum of Tregs for the management of wound pathologies should be further researched and more clinical trials are warranted to integrate Tregs into the standard clinical workflow of wound healing care.

## Conclusions

Skin injury triggers a highly complex cascade of healing processes aimed to mitigate tissue damage whilst restoring cutaneous integrity. During this temporally- and spatially-synchronized interplay between skin cells and immunocytes, Tregs play a crucial role. Their field of activity is multifaceted and ranges from preventing excessive inflammation and balancing immune homeostasis to facilitating wound healing and regenerating skin adnexa. Accordingly, Tregs’ absence echoes in cellular shifts and alterations in the cytokine (micro)environment. A comprehensive understanding of Tregs’ operational and functional diversity can, therefore, help decipher skin and wound pathologies. Ultimately, these insights may also be translated into clinically effective treatment modalities.

## Data Availability

Not applicable.

## Abbreviations

AA: Alopecia areata
Akt: Akt kinase
AREG: Amphiregulin
BATF: Basic leucine zipper ATF-like
BCL-2: B cell lymphoma- 2
bFGF: Basic fibroblast growth factor
BG-EGF: Heparin-binding EGF-like growth factor
Blimp1: B lymphocyte-induced maturation protein-1
CAR: Chimeric antigen receptors
Cas9: CRISPR-associated protein 9
CCL: CC-chemokine ligand
CCR: CC-chemokine receptor
CD: Cluster of Differentiation
CLA: Cutaneous lymphocyte-associated antigen
COL1A1: Type I collagen alpha 1
COL3A1: Type III collagen alpha 1
CRISPR: Clustered regularly interspaced short palindromic repeats
CTLA-4: Cytotoxic T lymphocyte protein 4
CXCL5: CXC motif chemokine 5
ECM: Extracellular cell matrix
EGF: Epidermal growth factor
EGFR: Epidermal growth factor receptor
EPF: Engrailed 1-lineage positive fibroblasts
FAS: Fas cell surface death receptor
FOXP3: Forkhead box protein P3
FuT7: α-1,3-Fucosyltransferase VII
GATA3: GATA binding protein 3
GITR: Glucocorticoid-induced TNFR-related protein
Gli1: GLI family zinc finger-1
GPIb: Glycoprotein Ib
Gr-1: Gamma response 1
HF: Hair follicle
HFSC: Hair follicle stem cell
HLA: Human leukocyte antigen
ICOS: Inducible co-stimulator
IFN-γ: Interferon gamma
IGF-1: Insulin-like growth factor 1
IKZF4: Ikaros family zinc finger 4
IL: Interleukin
IPEX: Immune dysregulation, polyendocrinopathy, enteropathy X-linked
KGF: Keratinocyte growth factor
ki-67: Kiel-67
KO: Knockout
Lgr: Leucine-rich repeat-containing G-protein coupled receptor
Ly6C: Lymphocyte antigen 6 complex
Met-ENK: Methionine enkephalin
MHC: Major histocompatibility complex
MMF: Mycophenolate mofetil
mTOR: Mechanistic target of rapamycin
NKG2D: Natural killer group 2D
P2X7R: Purinergic receptor P2X7
PD-1: Programmed cell death protein 1
PDGF: Platelet-derived growth factor
PENK: Proenkephalin
PI3K: Phosphoinositide-3-kinases
p-Tregs: Peripherally-derived regulatory T cells
Rnf20: Ring finger protein 20
RORα: RAR-related orphan receptor alpha
TBX21: T-box transcription factor 21
TCR: T cell receptor
Tfh-like: Nonlymphoid T follicular helper-like
TGF-α: Transforming growth factor-alpha
TGF-β: Transforming growth factor-beta
Th2: T helper cell type 2
TNF-α: Tumor necrosis factor-alpha
Tregs: Regulatory T cells
t-Tregs: Thymus-derived regulatory T cells
UVB: Ultraviolet B
VCA: Vascularized composite allotransplantation
VEGF: Vascular endothelial growth factor
α-SMA: α-Smooth muscle actin
